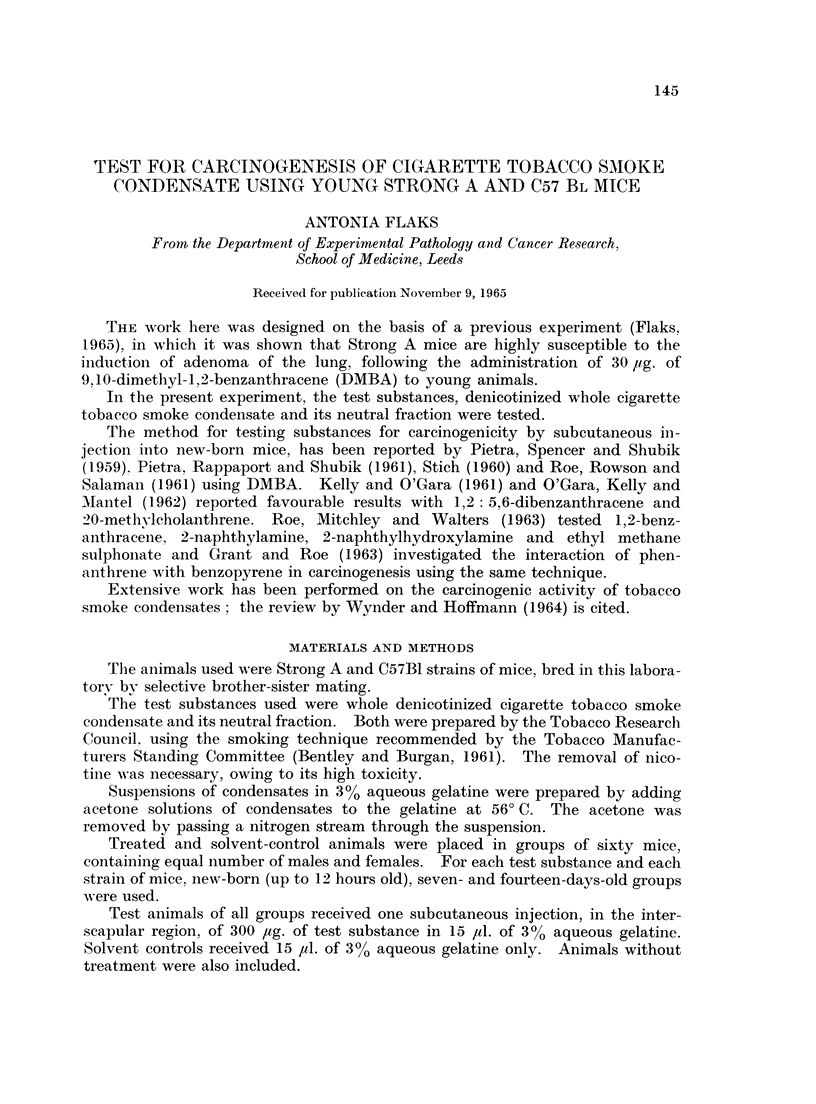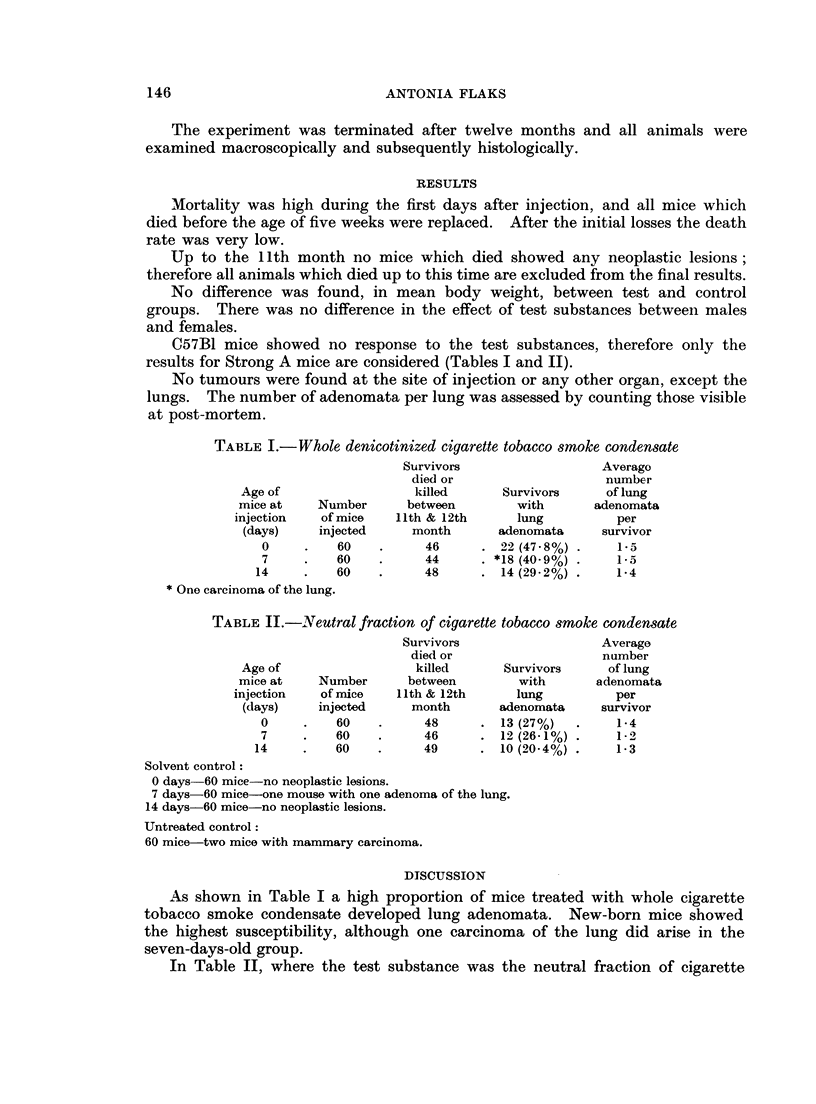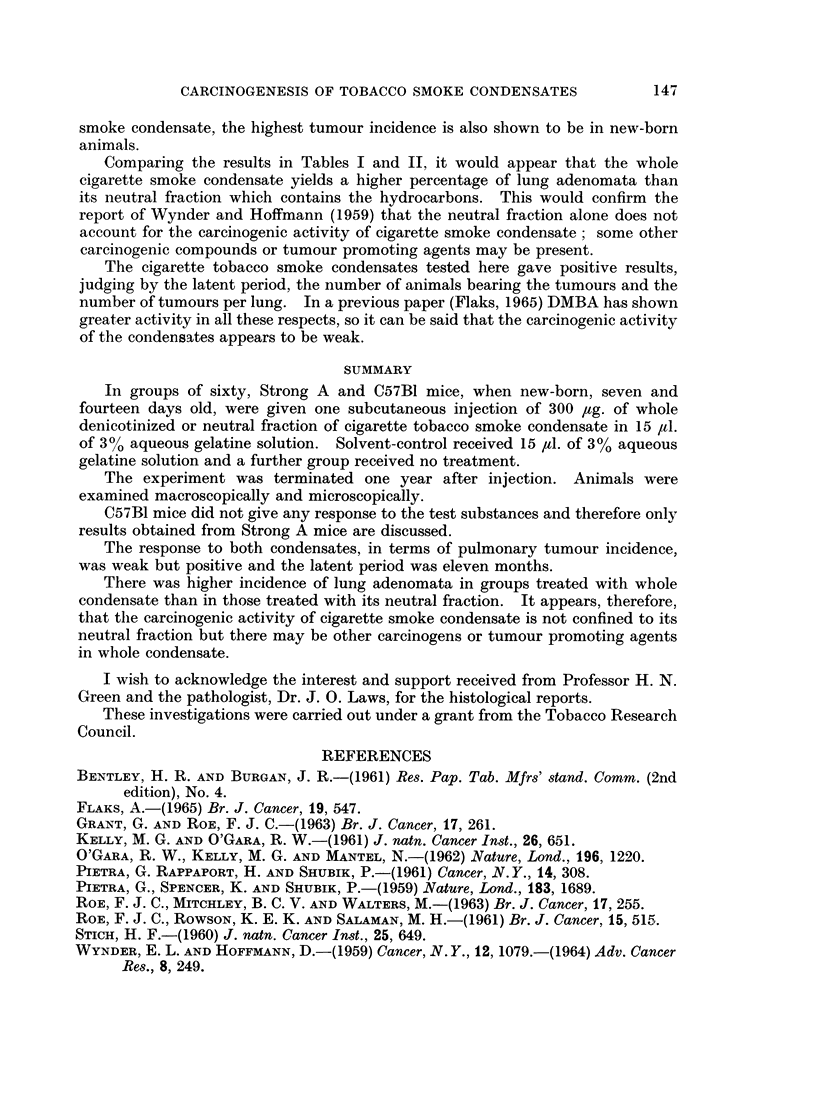# Test for carcinogenesis of cigarette tobacco smoke condensate using young strong A and C57 Bl mice.

**DOI:** 10.1038/bjc.1966.16

**Published:** 1966-03

**Authors:** A. Flaks


### TEST FOR CARCINOGENESIS OF CIGARETTE TOBACCO SMOKE CONDENSATE USING YOUNG STRONG A AND C57 BL MICE

ANTONIA FLAKS

*From the Department of Experimental Pathology and Cancer Research, School of Medicine, Leeds*

Received for publication November 9, 1965

THE work here was designed on the basis of a previous experiment (Flaks, 1965), in which it was shown that Strong A mice are highly susceptible to the induction of adenoma of the lung, following the administration of 30 $\mu$g. of 9,10-dimethyl-1,2-benzanthracene (DMBA) to young animals.

In the present experiment, the test substances, denicotinized whole cigarette tobacco smoke condensate and its neutral fraction were tested.

The method for testing substances for carcinogenicity by subcutaneous injection into new-born mice, has been reported by Pietra, Spencer and Shubik (1959), Pietra, Rappaport and Shubik (1961), Stich (1960) and Roe, Rowson and Salaman (1961) using DMBA. Kelly and O'Gara (1961) and O'Gara, Kelly and Mantel (1962) reported favourable results with 1,2 : 5,6-dibenzanthracene and 20-methylcholanthrene. Roe, Mitchley and Walters (1963) tested 1,2-benzanthracene, 2-naphthylamine, 2-naphthylhydroxylamine and ethyl methane sulphonate and Grant and Roe (1963) investigated the interaction of phenanthrene with benzopyrene in carcinogenesis using the same technique.

Extensive work has been performed on the carcinogenic activity of tobacco smoke condensates ; the review by Wynder and Hoffmann (1964) is cited.

### MATERIALS AND METHODS

The animals used were Strong A and C57Bl strains of mice, bred in this laboratory by selective brother-sister mating.

The test substances used were whole denicotinized cigarette tobacco smoke condensate and its neutral fraction. Both were prepared by the Tobacco Research Council, using the smoking technique recommended by the Tobacco Manufacturers Standing Committee (Bentley and Burgan, 1961). The removal of nicotine was necessary, owing to its high toxicity.

Suspensions of condensates in 3% aqueous gelatine were prepared by adding acetone solutions of condensates to the gelatine at 56° C. The acetone was removed by passing a nitrogen stream through the suspension.

Treated and solvent-control animals were placed in groups of sixty mice, containing equal number of males and females. For each test substance and each strain of mice, new-born (up to 12 hours old), seven- and fourteen-days-old groups were used.

Test animals of all groups received one subcutaneous injection, in the interscapular region, of 300 $\mu$g. of test substance in 15 $\mu$l. of 3% aqueous gelatine. Solvent controls received 15 $\mu$l. of 3% aqueous gelatine only. Animals without treatment were also included.

The experiment was terminated after twelve months and all animals were examined macroscopically and subsequently histologically.

### RESULTS

Mortality was high during the first days after injection, and all mice which died before the age of five weeks were replaced. After the initial losses the death rate was very low.

Up to the 11th month no mice which died showed any neoplastic lesions; therefore all animals which died up to this time are excluded from the final results.

No difference was found, in mean body weight, between test and control groups. There was no difference in the effect of test substances between males and females.

C57Bl mice showed no response to the test substances, therefore only the results for Strong A mice are considered (Tables I and II).

No tumours were found at the site of injection or any other organ, except the lungs. The number of adenomata per lung was assessed by counting those visible at post-mortem.

TABLE I.—*Whole denicotinized cigarette tobacco smoke condensate*

| Age of mice at injection (days) | Number of mice injected | Survivors died or killed between 11th & 12th month | Survivors with lung adenomata | Averago number of lung adenomata per survivor |
|---|---|---|---|---|
| 0 | 60 | 46 | 22 (47·8%) | 1·5 |
| 7 | 60 | 44 | *18 (40·9%) | 1·5 |
| 14 | 60 | 48 | 14 (29·2%) | 1·4 |

* One carcinoma of the lung.

TABLE II.—*Neutral fraction of cigarette tobacco smoke condensate*

| Age of mice at injection (days) | Number of mice injected | Survivors died or killed between 11th & 12th month | Survivors with lung adenomata | Average number of lung adenomata per survivor |
|---|---|---|---|---|
| 0 | 60 | 48 | 13 (27%) | 1·4 |
| 7 | 60 | 46 | 12 (26·1%) | 1·2 |
| 14 | 60 | 49 | 10 (20·4%) | 1·3 |

Solvent control:
0 days—60 mice—no neoplastic lesions.
7 days—60 mice—one mouse with one adenoma of the lung.
14 days—60 mice—no neoplastic lesions.

Untreated control:
60 mice—two mice with mammary carcinoma.

### DISCUSSION

As shown in Table I a high proportion of mice treated with whole cigarette tobacco smoke condensate developed lung adenomata. New-born mice showed the highest susceptibility, although one carcinoma of the lung did arise in the seven-days-old group.

In Table II, where the test substance was the neutral fraction of cigarette smoke condensate, the highest tumour incidence is also shown to be in new-born animals.

Comparing the results in Tables I and II, it would appear that the whole cigarette smoke condensate yields a higher percentage of lung adenomata than its neutral fraction which contains the hydrocarbons. This would confirm the report of Wynder and Hoffmann (1959) that the neutral fraction alone does not account for the carcinogenic activity of cigarette smoke condensate ; some other carcinogenic compounds or tumour promoting agents may be present.

The cigarette tobacco smoke condensates tested here gave positive results, judging by the latent period, the number of animals bearing the tumours and the number of tumours per lung. In a previous paper (Flaks, 1965) DMBA has shown greater activity in all these respects, so it can be said that the carcinogenic activity of the condensates appears to be weak.

SUMMARY

In groups of sixty, Strong A and C57Bl mice, when new-born, seven and fourteen days old, were given one subcutaneous injection of 300 $\mu$g. of whole denicotinized or neutral fraction of cigarette tobacco smoke condensate in 15 $\mu$l. of 3% aqueous gelatine solution. Solvent-control received 15 $\mu$l. of 3% aqueous gelatine solution and a further group received no treatment.

The experiment was terminated one year after injection. Animals were examined macroscopically and microscopically.

C57Bl mice did not give any response to the test substances and therefore only results obtained from Strong A mice are discussed.

The response to both condensates, in terms of pulmonary tumour incidence, was weak but positive and the latent period was eleven months.

There was higher incidence of lung adenomata in groups treated with whole condensate than in those treated with its neutral fraction. It appears, therefore, that the carcinogenic activity of cigarette smoke condensate is not confined to its neutral fraction but there may be other carcinogens or tumour promoting agents in whole condensate.

I wish to acknowledge the interest and support received from Professor H. N. Green and the pathologist, Dr. J. O. Laws, for the histological reports.

These investigations were carried out under a grant from the Tobacco Research Council.

### REFERENCES

Bentley, H. R. and Burgan, J. R.—(1961) *Res. Pap. Tab. Mfrs' stand. Comm.* (2nd edition), No. 4.
Flaks, A.—(1965) *Br. J. Cancer*, **19**, 547.
Grant, G. and Roe, F. J. C.—(1963) *Br. J. Cancer*, **17**, 261.
Kelly, M. G. and O'Gara, R. W.—(1961) *J. natn. Cancer Inst.*, **26**, 651.
O'Gara, R. W., Kelly, M. G. and Mantel, N.—(1962) *Nature, Lond.*, **196**, 1220.
Pietra, G. Rappaport, H. and Shubik, P.—(1961) *Cancer, N.Y.*, **14**, 308.
Pietra, G., Spencer, K. and Shubik, P.—(1959) *Nature, Lond.*, **183**, 1689.
Roe, F. J. C., Mitchley, B. C. V. and Walters, M.—(1963) *Br. J. Cancer*, **17**, 255.
Roe, F. J. C., Rowson, K. E. K. and Salaman, M. H.—(1961) *Br. J. Cancer*, **15**, 515.
Stich, H. F.—(1960) *J. natn. Cancer Inst.*, **25**, 649.
Wynder, E. L. and Hoffmann, D.—(1959) *Cancer, N.Y.*, **12**, 1079.—(1964) *Adv. Cancer Res.*, **8**, 249.